# The use of multi-criteria decision making models in evaluating anesthesia method options in circumcision surgery

**DOI:** 10.1186/s12911-017-0409-5

**Published:** 2017-01-23

**Authors:** Gulsah Hancerliogullari, Kadir Oymen Hancerliogullari, Emrah Koksalmis

**Affiliations:** 1 School of Management, Centre of Operational Research, Management Sciences & Information Systems, University of Southampton, Southampton, UK; 2 Department of Pediatric Surgery, Faculty of Medicine, Giresun University, Giresun, Turkey; 3 Department of Industrial Engineering, Faculty of Management, Istanbul Technical University, Istanbul, Turkey

**Keywords:** Circumcision surgery procedure, Anesthesia methods, Health care, Multi-criteria, Fuzzy, AHP, TOPSIS

## Abstract

**Background:**

Determining the most suitable anesthesia method for circumcision surgery plays a fundamental role in pediatric surgery. This study is aimed to present pediatric surgeons’ perspective on the relative importance of the criteria for selecting anesthesia method for circumcision surgery by utilizing the multi-criteria decision making methods.

**Methods:**

Fuzzy set theory offers a useful tool for transforming linguistic terms into numerical assessments. Since the evaluation of anesthesia methods requires linguistic terms, we utilize the fuzzy Analytic Hierarchy Process (AHP) and fuzzy Technique for Order Preference by Similarity to Ideal Solution (TOPSIS). Both mathematical decision-making methods are originated from individual judgements for qualitative factors utilizing the pair-wise comparison matrix. Our model uses four main criteria, eight sub-criteria as well as three alternatives. To assess the relative priorities, an online questionnaire was completed by three experts, pediatric surgeons, who had experience with circumcision surgery.

**Results:**

Discussion of the results with the experts indicates that time-related factors are the most important criteria, followed by psychology, convenience and duration. Moreover, general anesthesia with penile block for circumcision surgery is the preferred choice of anesthesia compared to general anesthesia without penile block, which has a greater priority compared to local anesthesia under the discussed main-criteria and sub-criteria.

**Conclusions:**

The results presented in this study highlight the need to integrate surgeons’ criteria into the decision making process for selecting anesthesia methods. This is the first study in which multi-criteria decision making tools, specifically fuzzy AHP and fuzzy TOPSIS, are used to evaluate anesthesia methods for a pediatric surgical procedure.

**Electronic supplementary material:**

The online version of this article (doi:10.1186/s12911-017-0409-5) contains supplementary material, which is available to authorized users.

## Background

**Fig. 1:**
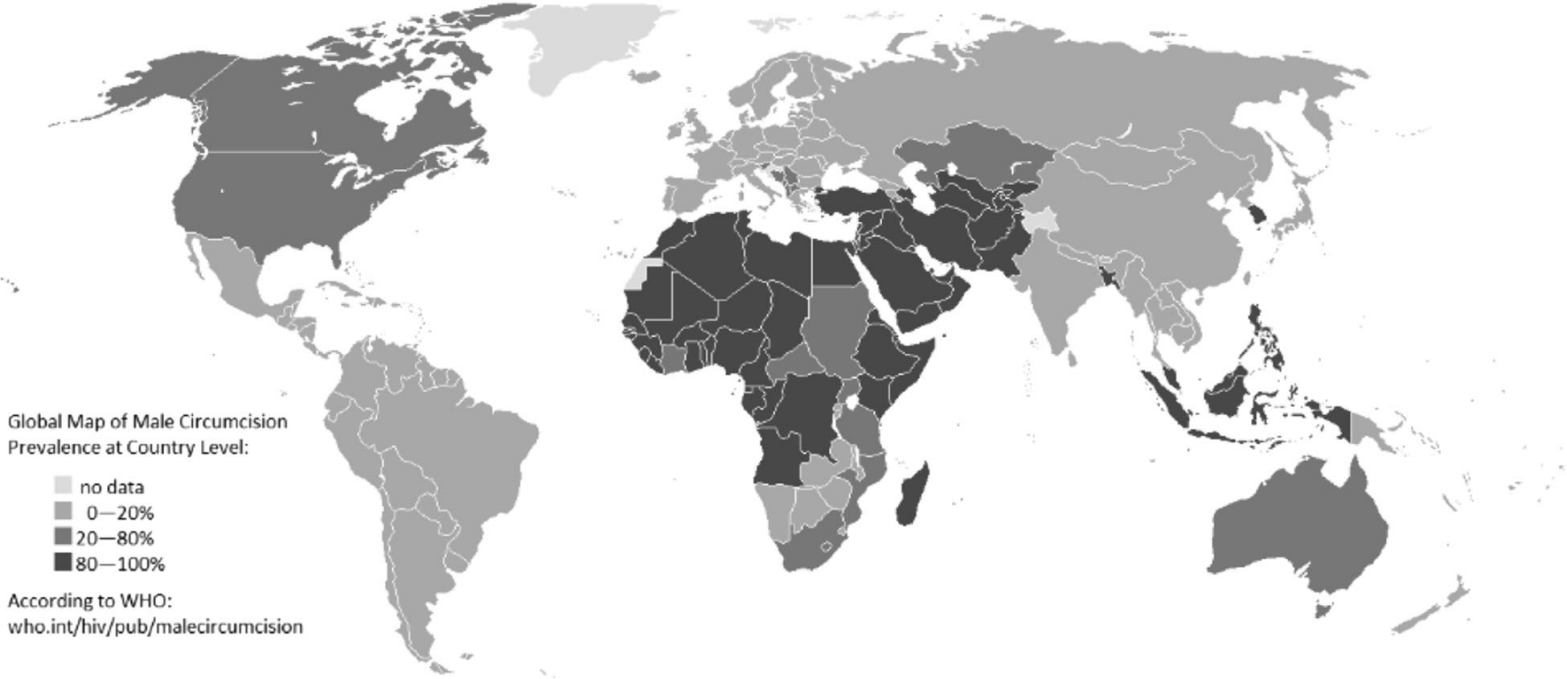
Global map of male circumcision prevalence at country level (source: http://who.int/hiv/pub/malecircumcision/globaltrends/en/)

Male circumcision, which is the surgical removal of the skin covering the tip of the penis, is the most commonly performed surgical procedure during the newborn period in certain parts of the world, including the United States [1–3]. It is performed with the use of devices such as the plastibell, the mogan clamp, or the gomco clamp. With all these devices, the same procedure is followed. Before the surgery begins, the genital area is thoroughly cleaned using a surgical scrub preparation. The doctor examines the patient’s penis to ensure he has no conditions which contraindicate circumcision [4]. Then, anesthesia is induced when the surgical team is completely prepared to begin. The doctor pulls the foreskin from the penis. Clipping is performed in, where the foreskin of the penis to the head grows. Finally, the foreskin is cut off with a scalpel or a special device. Doctor, perhaps, need to take steps to reduce bleeding or sew up the area to crop [5, 6]. This procedure is usually performed in an outpatient setting without the need to stay in hospital. Approximately 55 to 65% of all newborn boys are circumcised in the United States [7]. The prevalence of circumcision refers to the percentage of males in a given population who have been circumcised. In 2007, the World Health Organization (WHO) estimated that globally one-third of males aged 15 years and over are circumcised [8]. Figure 1 shows estimated country-level prevalence of male circumcision.

Several researchers have indicated the reliability and safety of anesthesia methods in eliminating the pain associated with circumcision [9–13]. Pediatric surgeons face with different alternatives while selecting an appropriate anesthesia method to apply in the circumcision procedure. The decision is complex since there are several factors affecting the operations. The alternatives of anesthesia methods performed include general anesthesia with penile block, without penile block, and local anesthesia. However, investigations have not been conducted to determine which anesthesia method is most effective under multiple conflicting criteria. Selection of a suitable anesthesia method depends on several factors. Deciding the appropriate method is a real concern since selection of an inappropriate method may threaten patients’ lives and lead to loss of resources and time. Nevertheless, physicians may ignore the defined criteria, sub-criteria and particular patient’s values, and select the anesthesia method that they usually apply or are good at. Hence, a multi-criteria evaluation approach is required. Evaluation of anesthesia methods in a surgery is a multi-criteria decision making (MCDM) problem since it involves many conflicting criteria. Moreover, the decision matrices that are used usually include vague and uncertain data. There are several approaches to handle multi-criteria problems in the literature. A variety of decision making approaches and tools are available to support health care and medical decision making.

The interest in healthcare multi-criteria decision making is very recent as the first studies were published in 1990s, and majority was published since 2000. The methodologies adopted in healthcare multi-criteria decision analysis are reviewed [14]. Several research studies discuss the use of AHP across a broad range of applications in health care and medical decision making. A tutorial is provided on the use of AHP in medical decision making [15]. The applicability of AHP for medical and hospital decision support is reviewed [16]. An extended literature review of AHP for important problems in medical and health care decision making are presented [17]. Furthermore, AHP has been proposed for its use in medical diagnosis, for the evaluation and selection of medical treatments and therapies, for organ transplant eligibility and allocation decisions [18–29].

In addition to AHP, several research studies discuss the use of TOPSIS, which is also another widely used multi-criteria decision making methods, across a broad range of applications in health care and medical decision making. An extended state-of-art survey of TOPSIS applications and methodologies including health and safety management are presented. TOPSIS has also been used in evaluating treatment and prevention options, and analyzing service quality in health care. A comprehensive performance analyses of health care for children using TOPSIS method are provided [30–38].

Although there have been several applications of AHP and TOPSIS methods in health care, no applications of fuzzy AHP and fuzzy TOPSIS methodologies for the evaluation of anesthesia methods was found in the literature. The purpose of this paper is to provide the use of multi-attribute decision making models in evaluating anesthesia methods in circumcision procedure. To our knowledge, this is the first study in which multi-criteria decision making tools, specifically fuzzy AHP and fuzzy TOPSIS, are used to evaluate the anesthesia methods for a surgery in literature.

## Methods

### Alternative anesthesia methods and criteria for evaluation

This study aims to select the most suitable alternative among the following three anesthesia methods in circumcision surgery procedure: General anesthesia without penile block (A1), general anesthesia with penile block (A2), local anesthesia (A3).

Local anesthesia is defined as loss of sensation in a circumscribed area of the body without inducing loss of consciousness. It is an anesthetic drug, which can be given as a shot, spray, or ointment, numbs only to a small, specific area of the body. Local anesthesia lasts for a short period of time and is often used for minor outpatient procedures [4, 39]. According to some researchers, circumcision is best done using a local anesthetic, especially early in infancy when the infant is less mobile [40]. For neonatal circumcision, it is stated that since local anesthesia is simple to perform, it shows greater efficacy compared to other anesthesia methods [41].

General anesthesia differs from local anesthesia, and it makes and keeps a person completely unconscious (or ‘asleep’) during the operation, with no awareness or memory of the surgery. General anesthesia is necessary for some surgical procedures since it may be safer or more comfortable for you to be unconscious. It will either be given as a liquid injected into your veins through a cannula or gas which you breathe in through a mask [42]. Surgical procedures in children, such as postneonatal circumcision, are usually performed with the patient under general anesthesia [43].

The first main criterion, denoted with C1, is convenience, which consists of two sub-criteria convenience for patient and doctor. The physiological structure, the age and the history of the patient are effective for the decision of which anesthesia method to choose. Therefore, these factors are taken into account under the C11, convenience for patient, sub-criteria. Due to the resource availability and some doctors having more experience on a specific method compared to the others; doctors may tend to choose the anesthesia method which they are in favor and good at. These cases are taken into account under the C12, convenience for doctor.

The second main criterion, denoted with C2, is reliability, which consists of two sub-criteria, condition of penis and vital function. Some patients are born with a common condition called hypospadias, where the urinary opening is not at the usual location on the head of the penis. Moreover, some patients have penis anomalies such as double hole; in which, general anesthesia methods are preferred compared to the local anesthesia. Therefore, these factors are taken into account under the C21, condition of penis. For the cases where the patients have problems in respiratory tract, heart functions, lung functions, in other words, for the medical conditions where narcosis is not suitable, or may have side effects or threat the vital functions, the local anesthesia method is more preferred. Hence, these cases are taken into account under the C22, vital function.

The third main criterion, denoted with C3, is duration, which consists of two sub-criteria, duration of operation, duration of recovery. Compared to the general anesthesia methods, the local anesthesia takes less time and this factor is considered under the C31, duration of operation. The patient may have to undergo a prolonged stay in hospital after general anesthesia due to the effect of narcosis; on the other hand, the patient can be discharged shortly after undergoing a local anesthetic. However, the process of the recovery of the penis is similar in both general anesthesia and local anesthesia methods, and this is considered under the C32, duration of recovery.

The forth main criterion, denoted with C4, is psychology, which consists of two sub-criteria: psychology of patient and psychology of parent. In addition to the physical damage, the circumcision affects the brain; the fact of being circumcised can cause distress, resentment, anger and depression. A local anesthetic is preferred to numb the area so there is less pain and risk of injury to the penis while the baby is still awake. As children get older, they become more aware of their sexual organs, so there are more psychological impacts associated with the surgery, and children become fearful. Therefore, in older children and adults, the procedure is commonly performed under general anesthesia [44]. The severe pain of circumcision and the changes to infant-maternal interaction observed after circumcision raise the question of the effects on the parents especially mothers. Some mothers clearly remember their son’s circumcision after many years as the worst day of their life [45]. If local anesthesia is given, which eliminates risk of general anesthesia, the child will feel pressure and movement but not pain; on the other hand, under general anesthesia, he will not experience any pain during the procedure [46]. Therefore, we take into account the sub-criteria, C41 and C42, psychology of patient, psychology of parent respectively.

Penile block, which represents 85% of anesthetic use in the USA, is a technique for blocking the dorsal nerves of the penis involving injection of anesthetic at the 10 and 2 o’clock positions at the base of the penis [47–49]. Numerous previous studies have evaluated the efficacy of penile block given under general anesthesia [50, 51]. It has been proposed that penile block alone provides successful intraoperative analgesia for circumcision in children. Moreover, it has been concluded that general anesthesia with penile block most reliably and safely eliminates the pain of circumcision [10]. Recently, it has been shown that general anesthesia with penile block is more effective for pediatric urological surgeries than standard general anesthesia, i.e., general anesthesia without penile block [52].

**Fig. 2:**
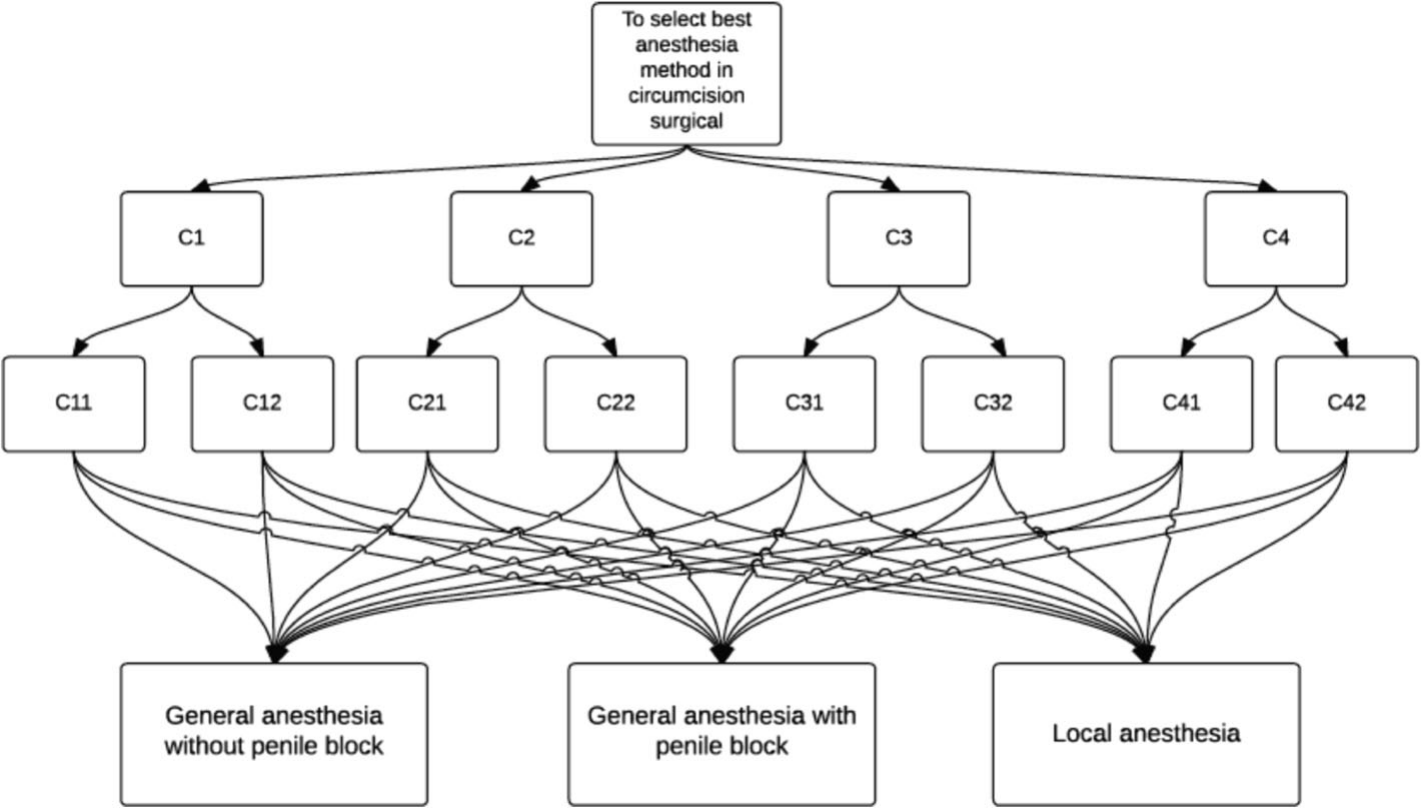
Hierarchy of criteria and alternatives

The surgeons have four main evaluation criteria for anesthesia methods in circumcision surgery procedure, which are convenience, psychology, reliability and duration. Each of these main criteria consists of two sub-criteria. The summary view of main evaluation criteria and sub-criteria for anesthesia methods can be seen in Table 1. Figure 2 illustrates the hierarchy between these criteria and alternatives.

**Table 1:** The evaluation criteria for anesthesia methods

Criteria	Sub-criteria
Convenience (C1)	Convenience for patient (C11)
	Convenience for doctor (C12)
Reliability (C2)	Condition of penis (C21)
	Vital function (C22)
Duration (C3)	Duration of anesthesia method (C31)
	Duration of recovery (C32)
Psychology (C4)	Psychology of parent (C41)
	Psychology of patient (C42)

### Fuzzy multi-attribute decision making methods

#### Fuzzy AHP

The analytic hierarchy process is a quantitative technique that deals with multi-attribute, multi-criteria, multi-person and multi-period problem hierarchically [53]. Even though the goal of AHP is to capture the expert’s experience, the traditional AHP still cannot fully reflect the vagueness in human thinking style. Therefore, fuzzy analytic hierarchy process (AHP) method, a fuzzy extension of AHP, was developed. In this paper, fuzzy AHP method [54], which is the extension of the conventional AHP method by integrating fuzzy comparison ratios, is used for the multi-criteria analysis.

In order to generate fuzzy weights and performance scores, geometric mean method is used. This method is used since it is easy to extend to the fuzzy case and guarantees a unique solution to the reciprocal comparison matrix. Recently, Buckley’s fuzzy AHP has been used in various applications such as selecting an ERP system for textile industry, urban industrial planning, academic personnel selection in maritime education, portfolio selection [55–59]. The steps of the fuzzy AHP can be summarized as follows:

- Step 1. Establish an expert team. The quality of the evaluation process depends on experts’ knowledge and experiences.
- Step 2. Determine the evaluation criteria and construct the hierarchy including alternatives. Literature or questionnaires assists the expert to determine evaluation criteria.
- Step 3. Construct pairwise comparison matrix and evaluate the relative importance of the criteria. The experts are expected to provide their judgments on the basis of their knowledge.
  For any expert the pairwise comparison matrix is given by Eq. (1) as:
  1 $$ {\tilde{C}}_k=\left[\begin{array}{ccc}\hfill 1\hfill & \hfill {\tilde{c}}_{12}\dots \hfill & \hfill {\tilde{c}}_{1n}\hfill \\ {}\hfill \vdots \hfill & \hfill \ddots \hfill & \hfill \vdots \hfill \\ {}\hfill {\tilde{c}}_{n1}\hfill & \hfill {\tilde{c}}_{n2}\cdots \hfill & \hfill 1\hfill \end{array}\right] $$
  where $n$ is the number of criteria, ${\tilde{C}}_k$ is a pairwise comparison matrix belongs to *k* ^th^ expert for *k* = 1, 2,.., *K*. Arithmetic mean is used to aggregate experts’ opinion as given in Eq. (2).
  2 $$ \tilde{C}=\frac{1}{K}\left({\tilde{C}}^1+{\tilde{C}}^2+\dots +{\tilde{C}}^K\right) $$
- Step 4. Transform the linguistic terms into triangular fuzzy numbers. The following linguistic terms provided in Table 2 are utilized for the evaluation procedure [60].

**Table 2:** Fuzzy evaluation scale for the weights

Linguistic terms	Triangular fuzzy scale
Equal (E)	(1,1,1)
Slightly Important (SI)	(1,1,3)
Fairly Important (FI)	(1,3,5)
Highly Important (HI)	(3,5,7)
Very Important (VI)	(5,7,9)
Extremely Important (EI)	(7,9,9)
- Step 5. Calculate the fuzzy weight matrix using Buckley’s method as follows using Eqs. (3) and (4).
  3 $$ {\tilde{r}}_i={\left({\tilde{c}}_{i1}\otimes {\tilde{c}}_{i2}\otimes \dots \otimes {\tilde{c}}_{in}\right)}^{\frac{1}{n}} $$
  4 $$ {\tilde{w}}_i={\tilde{r}}_i\otimes {\left({\tilde{r}}_1+{\tilde{r}}_2+\dots +{\tilde{r}}_n\right)}^{-1} $$
  where ${\tilde{r}}_i$ is the geometric mean of fuzzy comparison value, ${\tilde{w}}_i$ is the fuzzy weight of the *i* ^th^ criterion. After the fuzzy relative weight matrix is calculated, fuzzy numbers are defuzzied into crisp values using a common method, centroid method [61], and then apply the normalization procedure as provided in Eq. (5).
  5 $$ {w}_i=\frac{{\tilde{w}}_i}{{\displaystyle {\sum}_{j=1}^n}{\tilde{w}}_j}=\frac{L_i+{M}_i+{U}_i}{{\displaystyle {\sum}_{j=1}^n}{\tilde{w}}_j} $$
- Step 6. Check the consistency of the pairwise comparison matrices by calculating the consistency ratios.
- Step 7. Select the best alternative using the weights of criteria and alternatives.

### Fuzzy TOPSIS

A computationally simple, effective and one of the classic multi-criteria decision making methods, TOPSIS, was developed [62]. The methodology chooses an alternative with the shortest distance from the positive ideal solution (PIS) and the farthest distance from the negative ideal solution (NIS). Then, in order to handle uncertainties in decision making problems, it was extended to a fuzzy environment [63]. Recently, fuzzy TOPSIS has been used in various applications such as prioritizing the best sites for treated wastewater in stream, location selection for the faculty of a university, improvement of the general flood vulnerability approach, selection of the green suppliers for an electronic company [64–67]. In the following, the steps of the fuzzy TOPSIS are provided [68].

- Step 1. Define a decision matrix, D as in Eq. (6). Evaluate the ratings of the alternatives using the linguistic variables and determine the criteria weights.
  6 $$ D=\begin{array}{c}\hfill {A}_1\hfill \\ {}\hfill \dots \hfill \\ {}\hfill {A}_m\hfill \end{array}\left[\begin{array}{ccc}\hfill {x}_{11}\ \hfill & \hfill {x}_{1j}\dots \hfill & \hfill {x}_{1n}\hfill \\ {}\hfill \vdots \hfill & \hfill \ddots \hfill & \hfill \vdots \hfill \\ {}\hfill {x}_{m1}\hfill & \hfill {x}_{mj}\cdots \hfill & \hfill {x}_{mn}\hfill \end{array}\right] $$
  where *x*_*ij*_ may be crisp or fuzzy. If it is fuzzy, it is represented by a trapezoidal number as *x*_*ij*_ = (*a*_*ij*_, *b*_*ij*_, *c*_*ij*_, *d*_*ij*_). The fuzzy weights are described by Eq. (7).
  7 $$ {w}_j=\left({\alpha}_j,{\beta}_j,{\gamma}_j,{\delta}_j\right) $$
- Step 2. Normalize the decision matrix using the linear scale transformation as in Eq. (8):
  8 $$ {r}_{ij}=\left\{\begin{array}{c}\hfill {x}_{ij}/{x}_j^{*},\ \forall j,\ {x}_j is\ a\  benefit\  attribute\hfill \\ {}\hfill {x}_j^{-}/{x}_{ij},\forall j,\ {x}_j is\ a\  cost\  attribute\ \hfill \end{array}\right. $$
  Then, the normalized decision matrix in Eq. (6) can be written in Eq. (9) by applying Eq. (8) as follows:
  9 $$ D=\left[\begin{array}{ccc}\hfill {r}_{11}\hfill & \hfill {r}_{1j}\dots \hfill & \hfill {r}_{1n}\hfill \\ {}\hfill \vdots \hfill & \hfill \ddots \hfill & \hfill \vdots \hfill \\ {}\hfill {r}_{m1}\hfill & \hfill {r}_{mj}\dots \hfill & \hfill {r}_{mn}\hfill \end{array}\right] $$
  When *x*_*ij*_ is crisp, its corresponding *r*_*ij*_ must be crisp; when *x*_*ij*_ is fuzzy, its corresponding *r*_*ij*_ must be fuzzy.
  Let *x*_j_^*^ = (*a*_j_^*^, *b*_j_^*^, *c*_j_^*^, *d*_j_^*^), *x*_*j*_^−^ = (*a*_*j*_^−^, *b*_*j*_^−^, *c*_*j*_^−^, *d*_*j*_^−^), and ${\tilde{x}}_{ij}=\left({a}_{ij},\ {b}_{ij},{c}_{ij},{d}_{ij}\right)$. Replace the Eq. (8) by these fuzzy operations, then obtain Eq. (10):
  10 $$ {\tilde{r}}_{ij}=\left\{\begin{array}{c}\hfill {\tilde{x}}_{ij\ }\left(\div \right){\tilde{x}}_j^{*}=\left(\frac{a_{ij}}{d_j^{*}},\frac{b_{ij}}{c_j^{*}},\frac{c_{ij}}{b_j^{*}},\frac{d_{ij}}{a_j^{*}}\right),\ for\  benefit\  attributes\hfill \\ {}\hfill {\tilde{x}}_j^{-}\left(\div \right){\tilde{x}}_{ij} = \left(\frac{a_j^{-}}{d_{ij}},\frac{b_j^{-}}{c_{ij}},\frac{c_j^{-}}{b_{ij}},\frac{d_j^{-}}{a_{ij}}\right),\ for\  cost\  attributes\hfill \end{array}\right. $$
- Step 3. Construct the fuzzy weighted normalized decision matrix using Eq. (11):
  11 $$ {\tilde{v}}_{ij}={\tilde{r}}_{ij}\otimes {\tilde{w}}_j $$
  where $$ {\tilde{w}}_j=\left({\alpha}_j,{\beta}_j,{\gamma}_j,{\delta}_j\right) $$.
  When both *r*_*ij*_ and *w*_*j*_ are crisp, *v*_*ij*_ is crisp. On the other hand, when either *r*_*ij*_ or *w*_*j*_ (or both) are fuzzy, Eq. (11) can be rewritten as follows in Eq. (12):
  12 $$ {\tilde{v}}_{ij}=\left\{\begin{array}{c}\hfill \left(\frac{a_{ij}}{d_j^{*}}{\alpha}_j,\frac{b_{ij}}{c_j^{*}}{\beta}_j,\frac{c_{ij}}{b_j^{*}}{\gamma}_j,\frac{d_{ij}}{a_j^{*}}{\delta}_j\right),\ for\  benefit\  attributes\hfill \\ {}\hfill \left(\frac{a_j^{-}}{d_{ij}}{\alpha}_j,\frac{b_j^{-}}{c_{ij}}{\beta}_j,\frac{c_j^{-}}{b_{ij}}{\gamma}_j,\frac{d_j^{-}}{a_{ij}}{\delta}_j\right),\ for\  cost\  attributes\hfill \end{array}\right. $$
  The result of Eq. (12) can be summarized as a Ṽ = [*ṽ*_*ij*_]_*m* × *n*_ matrix in Eq. (13):
  13 $$ \tilde{V}=\left[\begin{array}{ccc}\hfill {\tilde{v}}_{11}\hfill & \hfill \dots \hfill & \hfill {\tilde{v}}_{1n}\hfill \\ {}\hfill \vdots \hfill & \hfill \ddots \hfill & \hfill \vdots \hfill \\ {}\hfill {\tilde{v}}_{m1}\hfill & \hfill \cdots \hfill & \hfill {\tilde{v}}_{mn}\hfill \end{array}\right] $$
- Step 4. Calculate the fuzzy positive ideal solution (FPIS, ṽ*) and the fuzzy negative ideal solution (FNIS, *ṽ* ^−^) as defined in the Eqs. (14) and (15) as follows:
  14 $$ {A}^{*}=\left[{\tilde{v}}_1^{*},\dots, {\tilde{v}}_n^{*}\right] $$
  15 $$ {A}^{-}=\left[{\tilde{v}}_1^{-},\dots, {\tilde{v}}_n^{-}\right] $$
  where *ṽ*_j_^*^ = *max*_*i*_ *ṽ*_*ij*_ and *ṽ*_*j*_^−^ = *min*_*i*_ *ṽ*_*ij*_.
- Step 5. Calculate the separation measures *S*_i_^*^ and *S*_*i*_^−^ that are defined in Eqs. (16) and (17) as follows:
  16 $$ {S}_i^{*}={\displaystyle {\sum}_{j=1}^n{D}_{ij}^{*},\ i=1,2,\dots,\ m.} $$
  17 $$ {S}_i^{-}={\displaystyle {\sum}_{j=1}^n{D}_{ij}^{-},\ i=1,2,\dots,\ m.} $$
  The difference measures, *D*_ij_^*^ and *D*_*ij*_^−^ for crisp data are defined in Eqs. (18) and (19) as follows :
  18 $$ {D}_{ij}^{*}=\left|{\tilde{v}}_{ij}-{\tilde{v}}_j^{*}\right| $$
  19 $$ {D}_{ij}^{-}=\left|{\tilde{v}}_{ij}-{\tilde{v}}_j^{-}\right| $$
- Step 6. Compute the closeness coefficient (*CC*_*i*_), which is defined to determine the ranking order of all alternatives. Once the crisp numbers *S*_i_^*^ and *S*_*i*_^−^, which can be combined, the *CC*_*i*_ of each alternative is calculated using the Eq. (20). Then, the alternatives are ranked in descending order of the *CC*_*i*_.
  20 $$ C{C}_i=\frac{S_i^{-}}{S_i^{*}+{S}_i^{-}} $$

### Participants

To assess the relative importance of aforementioned anesthesia method selection criteria, a questionnaire was designed, which is available as Additional file 1. An online questionnaire, which consists of the participants’ basic characteristics, demographic questions, was prepared based on the identified main criteria and sub-criteria. Participants were recruited in discussion forums of the pediatric surgeons by posting a link to the online-based questionnaire. All participants viewed a brief explanation of the study and their rights on the first page. The participants were experts, in other words pediatric surgeons, who had experience with circumcision procedure. They were asked to rate the relative priority of each criterion with other criterion at the same level within each level of the hierarchy. A pilot questionnaire was conducted with few pediatric surgeons; based on the input received, it was refined.

## Results

### Application: anesthesia methods in circumcision surgery

#### Fuzzy AHP

The steps of the methodology provided in the Methods section are applied for the problem.

- Step 1. The expert team consists of three experts, pediatric surgeons.
- Step 2. The evaluation criteria are determined, and have been provided in Table 1 and the hierarchy of the criteria and alternatives are provided in Fig. 2.
- Step 3. After several surveys have been conducted, experts determined the criteria weights by the pairwise comparison matrix. Constructed consensus matrices are provided in Table 3.

**Table 3:** Consensus matrices

Criteria	C1	C2	C3	C4		C1	C11	C12	C2	C21	C22		C3	C31	C32		C4	C41	C42
**C1**	E	1/VI	FI	1/HI		**C11**	E	HI	**C21**	E	1/VI		**C31**	E	VI		**C41**	E	1/FI
**C2**	VI	E	VI	HI		**C12**	1/HI	E	**C22**	VI	E		**C32**	1/VI	E		**C42**	FI	E
**C3**	1/FI	1/VI	E	1/HI															
**C4**	HI	1/HI	HI	E															
**C11**	**A1**	**A2**	**A3**		**C12**	**A1**	**A2**	**A3**	**C21**	**A1**	**A2**	**A3**		**C22**	**A1**	**A2**	**A3**		
**A1**	E	1/HI	FI		**A1**	E	1/HI	1/FI	**A1**	E	1/SI	HI		**A1**	E	SI	1/FI		
**A2**	HI	E	HI		**A2**	HI	E	VI	**A2**	SI	E	VI		**A2**	1/SI	E	HI		
**A3**	1/FI	1/HI	E		**A3**	FI	1/VI	E	**A3**	1/HI	1/VI	E		**A3**	FI	1/HI	E		
**C31**	**A1**	**A2**	**A3**		**C32**	**A1**	**A2**	**A3**	**C41**	**A1**	**A2**	**A3**		**C42**	**A1**	**A2**	**A3**		
**A1**	E	1/VI	1/VI		**A1**	E	1/HI	FI	**A1**	E	1/HI	SI		**A1**	E	1/FI	SI		
**A2**	VI	E	SI		**A2**	HI	E	VI	**A2**	HI	E	FI		**A2**	FI	E	FI		
**A3**	VI	1/SI	E		**A3**	1/FI	1/VI	E	**A3**	1//SI	1/FI	E		**A3**	1/SI	1/FI	E
- Step 4. The linguistic terms are transformed into triangular fuzzy numbers given in Table 2.
- Step 5. The final weights of the alternatives are calculated using Eqs. (3), (4) and (5). Illustrative examples for weights of sub-criteria C31 and C32 are given as follows:
  $$ {\tilde{r}}_{C31}={\left({\tilde{c}}_{C31C31}\otimes {\tilde{c}}_{C31C32}\right)}^{\frac{1}{2}} $$
  $$ {\tilde{r}}_{C31}={\left(\left(1,1,1\right)\otimes \left(5,7,9\right)\right)}^{\frac{1}{2}} $$
  $$ {\tilde{r}}_{C31}=\left(2.23,2.64,3\right) $$
  $$ {\tilde{r}}_{C32}={\left({\tilde{c}}_{C32C31}\otimes {\tilde{c}}_{C32C32}\right)}^{\frac{1}{2}} $$
  $$ {\tilde{r}}_{C32}={\left(1/\left(5,7,9\right)\otimes \left(1,1,1\right)\right)}^{\frac{1}{2}} $$
  $$ {\tilde{r}}_{C32}=\left(0.33,0.37,0.44\right) $$
  $$ {\tilde{w}}_{C31}={\tilde{r}}_{C31}\otimes {\left({\tilde{r}}_{C31}+{\tilde{r}}_{C32}\right)}^{-1} $$
  $$ {\tilde{w}}_{C31}=\left(2.23,2.64,3\right)\otimes {\left[\left(2.23,2.64,3\right)+\left(0.33,0.37,0.44\right)\right]}^{-1} $$
  $$ {\tilde{w}}_{C31}=\left(0.64,0.87,1.17\right) $$
  $$ {\tilde{w}}_{C32}={\tilde{r}}_{C32}\otimes {\left({\tilde{r}}_{C31}+{\tilde{r}}_{C32}\right)}^{-1} $$
  $$ {\tilde{w}}_{C32}=\left(0.33,0.37,0.44\right)\otimes {\left[\left(2.23,2.64,3\right)+\left(0.33,0.37,0.44\right)\right]}^{-1} $$
  $$ {\tilde{w}}_{C32}=\left(0.09,0.12,0.17\right) $$
  $$ {w}_{C31}=\frac{{\tilde{w}}_{C31}}{{\displaystyle {\sum}_{j=1}^2}{\tilde{w}}_{C3j}}=\frac{L_{C31}+{M}_{C31}+{U}_{C31}}{{\tilde{w}}_{C31}+{\tilde{w}}_{C32}} $$
  $$ {w}_{C31}=\frac{\left(0.64+0.87+1.17\right)}{\left(0.64+0.87+1.17+0.09+0.12+0.17\right)}=0.88 $$
  $$ {w}_{C32}=\frac{{\tilde{w}}_{C32}}{{\displaystyle {\sum}_{j=1}^2}{\tilde{w}}_{C3j}}=\frac{L_{C32}+{M}_{C32}+{U}_{C32}}{{\tilde{w}}_{C31}+{\tilde{w}}_{C32}} $$
  $$ {w}_{C32}=\frac{\left(0.09+0.12+0.17\right)}{\left(0.64+0.87+1.17+0.09+0.12+0.17\right)}=0.12 $$
  The similar calculation approach is applied for all pairwise comparisons. The final weights of the alternatives are provided in Table 4. An illustrative example for *W*_*A*3_ is provided as follows:

**Table 4:** Final weights

	C1		C2		C3		C4		
	0.08		0.62		0.05		0.25		
	C11	C12	C21	C22	C31	C32	C41	C42	
	0.83	0.17	0.14	0.86	0.88	0.12	0.30	0.70	W
A1	0.27	0.13	0.62	0.39	0.08	0.23	0.24	0.33	0.36
A2	0.87	0.80	0.88	0.63	0.57	0.88	0.78	0.73	0.69
A3	0.15	0.18	0.14	0.35	0.45	0.10	0.24	0.25	0.29

Consistency ratio (CR): 0.001 (values at 0.1 or below represent 90% or higher confidence level)

  $$ {W}_{A3}=0.08\times 0.83\times 0.15+0.08\times 0.17\times 0.18 + \dots +0.25\times 0.30\times 0.24+0.25\times 0.70\times 0.25=0.29 $$
- Step 6. The consistency ratios of the comparison matrices are checked.
- Step 7. According to Table 4, the best anesthesia method in circumcision surgery is Alternative 2, general anesthesia with penile block.

#### Fuzzy TOPSIS

The steps of the methodology provided in the Methods section are applied for the problem.

- Step 1. Experts assess the ratings of the alternatives, provided in Table 5, using a linguistic scale.

**Table 5:** Linguistic evaluation of alternatives by each expert

Expert 1	C1		C2		C3		C4	
	C11	C12	C21	C22	C31	C32	C41	C42
A1	VG	VG	F	G	VG	G	VG	G
A2	F	P	G	F	VG	P	F	F
A3	G	F	F	G	F	F	VG	F
Expert 2	C1		C2		C3		C4	
	C11	C12	C21	C22	C31	C32	C41	C42
A1	G	F	G	F	F	V	F	F
A2	VG	VG	VG	G	G	VG	VG	G
A3	F	G	P	F	G	F	F	G
Expert 3	C1		C2		C3		C4	
	C11	C12	C21	C22	C31	C32	C41	C42
A1	F	F	G	F	F	G	P	F
A2	G	G	VG	G	G	VG	F	G
A3	F	VG	F	F	F	F	P	F
  The details of the linguistic scale for the evaluation of the alternatives are provided in Table 6.

**Table 6:** Fuzzy evaluation scale for the alternatives

Linguistic terms	Triangular fuzzy scale
Poor (P)	(0,2.5,5)
Fair (F)	(2.5,5,7.5)
Good (G)	(5,7.5,10)
Very Good (VG)	(7,10,10)
- Step 2. The linguistic terms are converted into triangular fuzzy numbers. Then, normalized values are computed by dividing each value to 10 which is the largest upper value in the evaluation matrix. As an illustration, the normalized matrix of Expert 3 is provided in Table 7.

**Table 7:** Normalized fuzzy decision matrix of Expert 3

Normalized Matrix								
Expert 3	C1		C2		C3		C4	
	C11	C12	C21	C22	C31	C32	C41	C42
A1	(0.25,0.5,0.75)	(0.25,0.5,0.75)	(0.5,0.75,1)	(0.25,0.5,0.75)	(0.25,0.5,0.75)	(0.5,0.75,1)	(0,0.25,0.5)	(0.25,0.5,0.75)
A2	(0.5,0.75,1)	(0.5,0.75,1)	(0.7,1,1)	(0.5,0.75,1)	(0.5,0.75,1)	(0.7,1,1)	(0.25,0.5,0.75)	(0.5,0.75,1)
A3	(0.25,0.5,0.75)	(0.7,1,1)	(0.25,0.5,0.75)	(0.25,0.5,0.75)	(0.25,0.5,0.75)	(0.25,0.5,0.75)	(0,0.25,0.5)	(0.25,0.5,0.75)
- Step 3. Weighted normalized fuzzy decision matrix of Expert 3 is provided in Table 8.

**Table 8:** Weighted normalized fuzzy decision matrix of Expert 3

Expert 3	C1 (0.08)		C2 (0.66)		C3 (0.05)		C4 (0.21)	
	C11 (0.90)	C12 (0.10)	C21 (0.17)	C22 (0.83)	C31 (0.88)	C32 (0.12)	C41 (0.40)	C42 (0.60)
A1	(0.018,0.036,0.054)	(0.002,0.004,0.006)	(0.056,0.084,0.112)	(0.136,0.273,0.410)	(0.011,0.022,0.033)	(0.003,0.004,0.006)	(0,0.021,0.042)	(0.031,0.063,0.094)
A2	(0.036,0.054,0.072)	(0.004,0.006,0.008)	(0.078,0.112,0.112)	(0.273,0.410,0.547)	(0.022,0.033,0.044)	(0.004,0.006,0.006)	(0.021,0.042,0.063)	(0.063,0.094,0.126)
A3	(0.018,0.036,0.054)	(0.005,0.008,0.008)	(0.028,0.056,0.084)	(0.273,0.410,0.547)	(0.011,0.022,0.033)	(0.001,0.003,0.004)	(0,0.021,0.042)	(0.031,0.063,0.094)
  Note that, here, to determine the criteria weights of main criteria (C1, C2, etc.) and sub-criteria (C11, C12, etc.), which are presented in parentheses, we utilize the weights obtained from the fuzzy AHP method. For example, the criteria weights associated with the Expert 3 are calculated using the pairwise matrices of the expert provided in Table 9.

**Table 9:** Pairwise matrices of Expert 3

Criteria	C1	C2	C3	C4		C1	C11	C12	C2	C21	C22		C3	C31	C32		C4	C41	C42
**C1**	E	1/VI	FI	1/HI		C11	E	EI	C21	E	1/HI		C31	E	VI		C41	E	1/SI
**C2**	VI	E	EI	VI		C12	1/EI	E	C22	HI	E		C32	1/VI	E		C42	SI	E
**C3**	1/FI	1/EI	E	1/HI															
**C4**	HI	1/VI	HI	E															
**C11**	**A1**	**A2**	**A3**		**C12**	**A1**	**A2**	**A3**	**C21**	**A1**	**A2**	**A3**		**C22**	**A1**	**A2**	**A3**		
**A1**	E	1/VI	FI		A1	E	1/HI	1/FI	A1	E	1/SI	HI		A1	E	SI	1/FI		
**A2**	VI	E	HI		A2	HI	E	VI	A2	SI	E	VI		A2	1/SI	E	VI		
**A3**	1/FI	1/HI	E		A3	FI	1/VI	E	A3	1/HI	1/VI	E		A3	FI	1/VI	E		
**C31**	**A1**	**A2**	**A3**		**C32**	**A1**	**A2**	**A3**	**C41**	**A1**	**A2**	**A3**		**C42**	**A1**	**A2**	**A3**		
**A1**	E	1/VI	1/VI		A1	E	1/HI	FI	A1	E	1/HI	SI		A1	E	1/SI	SI		
**A2**	VI	E	SI		A2	HI	E	VI	A2	HI	E	SI		A2	SI	E	FI		
**A3**	VI	1/SI	E		A3	1/FI	1/VI	E	A3	1/SI	1/SI	E		A3	1/SI	1/FI	E
  Regarding to the Table 7, an illustrative example is given for the A3 under C31 as follows:
  $$ {\tilde{A}}_3^w={w}_{C3}\times {w}_{C31}\times {\tilde{A}}_3 $$
  $$ {\tilde{A}}_3^w=0.05\times 0.88\times \left(0.25,0.5,0.75\right) $$
  $$ {\tilde{A}}_3^w=\left(0.011,0.022,0.033\right) $$
- Step 4. The distances of each alternative from the fuzzy positive ideal solution and the fuzzy negative ideal solution for Expert 3 are given in Tables 10 and 11. An illustrative example is provided for the value given in the third row of the sixth column of Table 10 as follows:

**Table 10:** The distances of each alternative from the fuzzy positive ideal solution for Expert 3

FPIS									
Expert 3	C1		C2		C3		C4		CC*
	C11	C12	C21	C22	C31	C32	C41	C42	
A1	0.557	0.573	0.545	0.346	0.565	0.576	0.565	0.541	4.268
A2	0.546	0.574	0.519	0.346	0.558	0.574	0.553	0.523	4.194
A3	0.557	0.575	0.529	0.424	0.565	0.575	0.565	0.541	4.331

**Table 11:** The distances of each alternative from the fuzzy negative ideal solution for Expert 3

FNIS										
Expert 3	C1		C2		C3		C4		CC^−^	CC_i_
	C11	C12	C21	C22	C31	C32	C41	C42		
A1	0.022	0.004	0.035	0.246	0.014	0.002	0.016	0.039	0.378	0.081
A2	0.032	0.004	0.059	0.246	0.020	0.003	0.026	0.057	0.446	0.096
A3	0.022	0.002	0.050	0.171	0.014	0.003	0.016	0.039	0.317	0.068

  $$ FPIS,{A}_3^{*}=1/3{\left[{\left(0.011-1\right)}^2+{\left(0.022-1\right)}^2+{\left(0.033-1\right)}^2\right]}^{1/2} $$
  $$ FPIS,{A}_3^{*}=0.565 $$
  The value given in the third row of the sixth column of Table 11 is calculated as follows:
  $$ FNIS,{A}_3^{-}=1/3{\left[{0.011}^2+{0.022}^2+{0.033}^2\right]}^{1/2} $$
  $$ FNIS,{A}_3^{-}=0.014 $$
- Steps 5–6. Once the separation measures are determined, closeness coefficients are calculated, which are presented in Tables 10 and 11.
  The value of *CC* ^*^ (4.331) for Alternative 3, which is obtained by taking the sum of the third row, is provided in the last column of Table 10. Similarly, the value of *CC* ^−^(0.317) for Alternative 3, is provided by taking the sum of the related row, is in the last column of Table 11.
  The value of *CC*_*i*_ for Alternative 3,*CC*_*3*_, is calculated as follows:
  $$ C{C}_3=\frac{0.317}{4.331+0.317} $$
  $$ C{C}_3=0.068 $$

### Sensitivity analysis

The objective of the sensitivity analysis is to observe how sensitive that our choice is to potential alterations in criteria weights. To do so, it is assumed that experts change their preferences for the criteria, and we consider various scenarios. In the following, the steps of the sensitivity analysis can be summarized:

- Step 1. The calculation procedure provided in the Methods section is applied for each expert’s preferences.
- Step 2. Various weighting scenarios, which are provided in Table 12, are applied in order to find a joint decision matrix.

**Table 12:** Weighting scenarios

	Scenario 1	Scenario 2	Scenario 3	Scenario 4
Expert 1	40%	30%	30%	33.33%
Expert 2	30%	40%	30%	33.33%
Expert 3	30%	30%	40%	33.33%
- Step 3. Ranking scores with respect to each expert is computed.

**Fig. 3:**
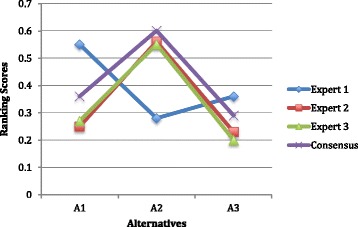
Rankings of alternatives obtained by fuzzy AHP

According to the Fig. 3, regarding the consensus decisions, the best anesthesia method alternative is the A2, which is general anesthesia with penile block. Moreover, except Expert 1, the rankings of the alternative anesthesia methods are similar.

**Fig. 4:**
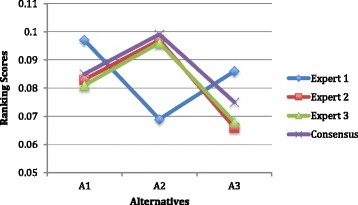
Rankings of alternatives obtained by fuzzy TOPSIS

The similar computation practice is applied to all experts’ preferences and weighting scenarios provided in the Methods section. Now, the rankings are represented through closeness coefficients which we provided a sample computational analysis in the Results section. The results are shown in Fig. 4, and the pattern is similar to Fig. 3.

Moreover, the consistency of responses and validity of the results are assessed based on the consistency ratio (CR), developed by Saaty, which enables to observe variations between the different pairwise comparisons. The CR value of all above pair-wise comparison matrix being 0.1 or below implies that the experts’ judgments are reasonable; values at 0.1 or above represent weak consistency [53]. The results indicate a good consistency with a CR of 0.01, which represents more than 90% confidence level (Table 4). Good consistency ratios infer that the responses expressed by experts are not arbitrary, and they are well thought responses.

## Discussion

In this paper, the evaluation of anesthesia methods is considered as a multi-criteria decision making problem which involves multiple and conflicting attributes. The decision matrices typically consist of vagueness and uncertainty, which are handled by the fuzzy sets. The results obtained by the fuzzy AHP are compared by the fuzzy TOPSIS method. The sensitivity analysis is conducted based on different scenarios of experts’ preferences; the results indicate that the ranking of the alternatives is insensitive to the probabilities in the criteria weights.

As expected, there are variations in the preferences through the multi-criteria decision making models. It is not straightforward for pediatric surgeons to make consistent decisions when faced with unfamiliar problems involving trade-offs between the advantages and disadvantages. The multi-criteria decision making methods were designed to help them make more informed choices in a step-by-step manner. The multi-criteria decision making methods, including AHP and TOPSIS, have been proposed for medical diagnosis, evaluation and selection of medical treatments and therapies; however, no studies have been done with the participation of pediatric surgeons. Discussion of the results with the expert participants confirmed that they consider these factors in the following order: first reliability, followed by psychology, convenience and duration.

Our research indicates that general anesthesia with penile block for circumcision surgery is the preferred choice of anesthesia compared to general anesthesia without penile block, which has a greater priority compared to local anesthesia under the discussed main-criteria and sub-criteria. Moreover, the reliability has a higher priority compared to the convenience, psychology and duration. Specifically, the vital function is more important than the condition of penis, as expected. The convenience for the patient is a more significant criterion than the convenience for the doctor. In addition, the decision maker should give more importance to the psychology of the patient compared to the psychology of the parent.

In order to offer feedback to facilitate the decision-making process, clinically practical decision-support tools have to be developed. Providing the strengths and limitations of each anesthesia method option definitely affects the patients’ and their parents’ decisions. Shared-decision making approach between patient and doctor should be applied in the evaluation and selection of therapy and treatment options. Our study provides a reference for a clinical decision-support system for pediatric surgeons, assisting them in multi-criteria decision making as well as providing details of the procedure to the patients with simple computerised processes.

### Limitations

This study has several limitations. Our sample was limited to few pediatric surgeons working in Turkey who had circumcision surgery experience. Future research in a more culturally diverse geographical region can be completed and compared with the results of this paper since preferences/experiences may change by country, tradition or socioeconomic level. As stated earlier, it is crucial to improve physician-patient discussions and adopt a shared-decision making approach. Therefore, preferences of patients (or their families) can also be observed similarly.

## Conclusions

To our knowledge, this is the first study to measure preferences of pediatric surgeons for anesthesia method selection for circumcision surgery. Deciding among the anesthesia methods is a complex decision problem due to the fact that each option has advantages and disadvantages. We presented the results of a study on the application of fuzzy AHP and fuzzy TOPSIS methodologies. A set of criteria for anesthesia method for circumcision were identified based on the literature review and inputs from experts, and organized into a rational hierarchical framework consisting of the four main criteria and eight sub-criteria.

For future research, further fuzzy decision making methods such as VIKOR, ELECTRE, PROMETHE, and ANP can be used, and their results can be compared with the results obtained in this paper. The model proposed in this research can also be applied to other multi-criteria decision making problems in medical or health care operations.

## Additional file

Additional file 1: Questionnaire. (PDF 485 kb)

## Abbreviations

AHP: Analytic hierarchy process
MCDM: Multi-criteria decision making
NIS: Negative ideal solution
PIS: Positive ideal solution
TOPSIS: Technique for order preference by similarity to ideal solution
UTI: Urinary tract infections
