# Supplementary material for: The use of multi-criteria decision making models in evaluating anesthesia method options in circumcision surgery
Source: BMC Med Inform Decis Mak. 2017 Jan 23;17:14. doi: 10.1186/s12911-017-0409-5 (PMC5260115; doi:10.1186/s12911-017-0409-5)
Supplement: Additional file 1: — Questionnaire. (PDF 485 kb) [file 12911_2017_409_MOESM1_ESM.pdf]

Dear Sir/Madam,

This survey will be the main source of data that will be evaluated when we investigate the weights of the factors that are effective in the process of choosing anesthesia methods used in circumcision surgery in Turkey.

In the success of the questionnaire and therefore of our work, your involvement and your complete answers have a great proposition. The answers you provide on the questionnaire will be kept strictly confidential and no data will be shared about the individual and will not be used for any purpose other than our work.

We will be pleased to share with you the findings we obtained as a result of our work in the relevant area. Thank you very much for your interest and help in your work.

Best Regards,

Asst.Prof.Gulsah Hancerliogullari
e-mail: gulsahhancerliogullari@gmail.com

Factors Affecting the Preference of Anesthesia Methods in Circumcision Surgery

1. Age : …………..
2. Gender : ☐ Male ☐ Female
3. Institution: ☐ Military hospital ☐ Private hospital ☐ University hospital ☐ Educational research hospital ☐ Public hospital
4. For many years you have been working as a pediatric surgeon? : ……………………………
5. For many years, you have being performed circumcision surgery? : ……………………………
6. When you think of the last 3 years, on average how many circumcision surgery did you perform per year? : ……………………………
7. When you think of the last 3 years, on average how many general anesthesia with penile block did you perform per year? : ……………………………
8. When you think of the last 3 years, on average how many general anesthesia without penile block did you perform per year? : ……………………………
9. When you think of the last 3 years, on average how many general anesthesia with penile block did you perform per year? : ……………………………
10. When you think of the last 3 years, on average how many local anesthesia did you perform per year? : ……………………………

Main criteria & sub-criteria

Convenience related factors

- Convenience for patient
- Convenience for doctor

Reliability related factors

- Condition of penis
- Vital function

Duration related factors

- Duration of anesthesia method
- Duration of recovery

Psychology related factors

- Psychology of parent
- Psychology of patient

Pairwise comparison evaluation method

On the following pages, you will be asked to evaluate the effects of the factors that may affect "the choice of anesthesia methods used in circumcision surgery". During the mentioned evaluation; the factors will be compared on a pairwise basis and expressed on the scale given.

1. In the selection of anesthesia method for circumcision surgery, please judge the relative importance: How important is each element on the left (A) compared with each element on the right (B)?

Example

While evaluating the "convenience related factors" and "reliability related factors" in the first line, if I mark "HI" on the "convenience related factors " side, I would prefer to consider "convenience related factors " as "highly important" rather than "reliability related factors" when selecting the anesthesia method for circumcision surgery. Similarly, if you think that "convenience related factors" are equal to "reliability related factors", you should mark “E” in the middle.

A	Column A compared to Column B		Equal importance level		Column B compared to Column A	B

E= Equal SI=Slightly Important FI=Fairly Important HI=Highly Important
VI= Very Important EI=Extremely Important

Convenience		EI	VI	HI	FI	SI	E	SI	FI	HI	VI	EI		Reliability
Convenience		EI	VI	HI	FI	SI	E	SI	FI	HI	VI	EI		Duration
Convenience		EI	VI	HI	FI	SI	E	SI	FI	HI	VI	EI		Psychology
Reliability		EI	VI	HI	FI	SI	E	SI	FI	HI	VI	EI		Duration
Reliability		EI	VI	HI	FI	SI	E	SI	FI	HI	VI	EI		Psychology

Duration		EI	VI	HI	FI	SI	E	SI	FI	HI	VI	EI		Psychology

2. In the selection of anesthesia method for circumcision surgery, when "convenience related factors" are considered only, please judge the relative importance: How important is each element on the left (A) compared with each element on the right (B)?

A	Column A compared to Column B		Equal importance level		Column B compared to Column A	B

E= Equal SI=Slightly Important FI=Fairly Important HI=Highly Important
VI= Very Important EI=Extremely Important

Convenience for patient		EI	VI	HI	FI	SI	E	SI	FI	HI	VI	EI		Convenience for doctor

3. In the selection of anesthesia method for circumcision surgery, when "reliability related factors" are considered only, please judge the relative importance: How important is each element on the left (A) compared with each element on the right (B)?

A	Column A compared to Column B		Equal importance level		Column B compared to Column A	B

E= Equal SI=Slightly Important FI=Fairly Important HI=Highly Important
VI= Very Important EI=Extremely Important

Condition of penis		EI	VI	HI	FI	SI	E	SI	FI	HI	VI	EI		Vital function

4. In the selection of anesthesia method for circumcision surgery, when "duration related factors" are considered only, please judge the relative importance: How important is each element on the left (A) compared with each element on the right (B)?

A	Column A compared to Column B		Equal importa nce level		Column B compared to Column A	B

E= Equal SI=Slightly Important FI=Fairly Important HI=Highly Important
VI= Very Important EI=Extremely Important

Duration of anesthesia method		EI	VI	HI	FI	SI	E	SI	FI	HI	VI	EI		Duration of recovery

5. In the selection of anesthesia method for circumcision surgery, when "psychology related factors" are considered only, please judge the relative importance: How important is each element on the left (A) compared with each element on the right (B)?

A	Column A compared to Column B		Equal importance level		Column B compared to Column A	B

E= Equal SI=Slightly Important FI=Fairly Important HI=Highly Important
VI= Very Important EI=Extremely Important

Psychology of parent		EI	VI	HI	FI	SI	E	SI	FI	HI	VI	EI		Psychology of patient

A	Column A compared to Column B		Equal importance level		Column B compared to Column A	B

E= Equal SI=Slightly Important FI=Fairly Important HI=Highly Important
VI= Very Important EI=Extremely Important

6. Compare the priorities of the following anesthesia methods in terms of convenience for patient.

General anesthesia without penile block				EI	VI	HI	FI	SI	E	SI	FI	HI	VI	EI				General anesthesia with penile block
General anesthesia without penile block				EI	VI	HI	FI	SI	E	SI	FI	HI	VI	EI				Local anesthesia
General anesthesia with penile block				EI	VI	HI	FI	SI	E	SI	FI	HI	VI	EI				Local anesthesia

7. Compare the priorities of the following anesthesia methods in terms of convenience for doctor.

General anesthesia without penile block				EI	VI	HI	FI	SI	E	SI	FI	HI	VI	EI				General anesthesia with penile block
General anesthesia without penile block				EI	VI	HI	FI	SI	E	SI	FI	HI	VI	EI				Local anesthesia
General anesthesia with penile block				EI	VI	HI	FI	SI	E	SI	FI	HI	VI	EI				Local anesthesia

8. Compare the priorities of the following anesthesia methods in terms of condition of penis.

General anesthesia without penile block					EI	VI	HI	FI	SI	E	SI	FI	HI	VI	EI				General anesthesia with penile block
General anesthesia without penile block					EI	VI	HI	FI	SI	E	SI	FI	HI	VI	EI				Local anesthesia
General anesthesia with penile block					EI	VI	HI	FI	SI	E	SI	FI	HI	VI	EI				Local anesthesia

9. Compare the priorities of the following anesthesia methods in terms of vital function.

General anesthesia without penile block					EI	VI	HI	FI	SI	E	SI	FI	HI	VI	EI				General anesthesia with penile block
General anesthesia without penile block					EI	VI	HI	FI	SI	E	SI	FI	HI	VI	EI				Local anesthesia
General anesthesia with penile block					EI	VI	HI	FI	SI	E	SI	FI	HI	VI	EI				Local anesthesia

10. Compare the priorities of the following anesthesia methods in terms of duration of anesthesia method.

General anesthesia without penile block				EI	VI	HI	FI	SI	E	SI	FI	HI	VI	EI				General anesthesia with penile block
General anesthesia without penile block				EI	VI	HI	FI	SI	E	SI	FI	HI	VI	EI				Local anesthesia
General anesthesia with penile block				EI	VI	HI	FI	SI	E	SI	FI	HI	VI	EI				Local anesthesia

11. Compare the priorities of the following anesthesia methods in terms of duration of recovery.

General anesthesia without penile block				EI	VI	HI	FI	SI	E	SI	FI	HI	VI	EI				General anesthesia with penile block
General anesthesia without penile block				EI	VI	HI	FI	SI	E	SI	FI	HI	VI	EI				Local anesthesia
General anesthesia with penile block				EI	VI	HI	FI	SI	E	SI	FI	HI	VI	EI				Local anesthesia

12. Compare the priorities of the following anesthesia methods in terms of psychology of parent.

General anesthesia without penile block				EI	VI	HI	FI	SI	E	SI	FI	HI	VI	EI				General anesthesia with penile block
General anesthesia without penile block				EI	VI	HI	FI	SI	E	SI	FI	HI	VI	EI				Local anesthesia
General anesthesia with penile block				EI	VI	HI	FI	SI	E	SI	FI	HI	VI	EI				Local anesthesia

13. Compare the priorities of the following anesthesia methods in terms of psychology of patient.

General anesthesia without penile block				EI	VI	HI	FI	SI	E	SI	FI	HI	VI	EI				General anesthesia with penile block
General anesthesia without penile block				EI	VI	HI	FI	SI	E	SI	FI	HI	VI	EI				Local anesthesia
General anesthesia with penile block				EI	VI	HI	FI	SI	E	SI	FI	HI	VI	EI				Local anesthesia
